# Long-term chloride concentrations in North American and European freshwater lakes

**DOI:** 10.1038/sdata.2017.101

**Published:** 2017-08-08

**Authors:** Hilary A. Dugan, Jamie C. Summers, Nicholas K. Skaff, Flora E. Krivak-Tetley, Jonathan P. Doubek, Samantha M. Burke, Sarah L. Bartlett, Lauri Arvola, Hamdi Jarjanazi, János Korponai, Andreas Kleeberg, Ghislaine Monet, Don Monteith, Karen Moore, Michela Rogora, Paul C. Hanson, Kathleen C. Weathers

**Affiliations:** 1 Center for Limnology, University of Wisconsin-Madison, 680 N Park St, Madison Wisconsin, 53706, USA; 2 Cary Institute of Ecosystem Studies, Box AB, Millbrook, New York 12545, USA; 3 Queen’s University, 99 University Ave, Kingston, Ontario, Canada K7L 3N6; 4 Department of Fisheries and Wildlife, Michigan State University, 13 Natural Resources Building, East Lansing, Michigan 48824, USA; 5 Department of Biological Sciences, Dartmouth College, 78 College Street, Hanover, New Hampshire 03755, USA; 6 Department of Biological Sciences, Virginia Tech, 926 West Campus Drive, Blacksburg, Virginia 24061, USA; 7 University of Waterloo, 200 University Ave. W, Waterloo, Ontario, Canada N2L 3G1; 8 School of Freshwater Sciences, University of Wisconsin-Milwaukee, 600 E Greenfield Ave, Milwaukee, Wisconsin 53204, USA; 9 Lammi Biological Station, University of Helsinki, FI-16900 Lammi, Finland; 10 Ontario Ministry of the Environment and Climate Change, Environmental Monitoring and Reporting Branch, 125 Resources Rd., Toronto, Ontario, Canada M9P 3V6; 11 MTA—PE Limnoecology Research Group, Egyetem u. 10, 8200 Veszprém, Hungary; 12 Department of Chemistry and Environmental Sciences, University of West Hungary, Károly Gáspár tér 4, 9700 Szombathely, Hungary; 13 Leibniz-Institute of Freshwater Ecology and Inland Fisheries, Müggelseedamm 301, D-12587 Berlin, Germany; 14 CARRTEL, INRA (French National Institute for Agronomical Research), 74203 Thonon-les-Bains, France; 15 NERC Centre for Ecology and Hydrology, Lancaster Environment Centre, Library Avenue, Bailrigg, Lancaster LA1 4AP, UK; 16 New York City Department of Environmental Protection, 71 Smith Ave., Kingston, New York 12401, USA; 17 CNR Institute of Ecosystem Study, L.go Tonolli 50, I-28922 Verbania Pallanza, Italy

**Keywords:** Freshwater ecology, Environmental chemistry, Limnology, Hydrology

## Abstract

Anthropogenic sources of chloride in a lake catchment, including road salt, fertilizer, and wastewater, can elevate the chloride concentration in freshwater lakes above background levels. Rising chloride concentrations can impact lake ecology and ecosystem services such as fisheries and the use of lakes as drinking water sources. To analyze the spatial extent and magnitude of increasing chloride concentrations in freshwater lakes, we amassed a database of 529 lakes in Europe and North America that had greater than or equal to ten years of chloride data. For each lake, we calculated climate statistics of mean annual total precipitation and mean monthly air temperatures from gridded global datasets. We also quantified land cover metrics, including road density and impervious surface, in buffer zones of 100 to 1,500 m surrounding the perimeter of each lake. This database represents the largest global collection of lake chloride data. We hope that long-term water quality measurements in areas outside Europe and North America can be added to the database as they become available in the future.

## Background & Summary

Recent analyses estimate there are 27 million naturally-formed lakes and human-constructed reservoirs (hereafter together referred to as lakes) on Earth with surface areas greater than 0.01 km^2^ (ref. 1). While these lakes account for less than four percent of Earth’s terrestrial land cover, they provide many important social and economic resources such as recreation, fisheries, irrigation, and energy production. Moreover, the vast majority^2^ of these lakes are freshwater lakes (salinity<1 g l^−1^), which are routinely used as drinking water sources. Therefore, it is critically important to protect the ecosystem services that lakes provide for humanity.

One important measure of water quality is the amount of salt, or salinity, in a lake. Chloride is often used as a measure of salinity because it is a highly conservative and highly soluble ion, is easily measured with high accuracy, and is a good proxy for salinity^3,4^. While lakes vary in their natural chloride concentrations due to geological factors^2^, changes in chloride levels can result from a response to climate^5^ and anthropogenic influences^6^. Anthropogenic sources of chloride include road salt, fertilizer, wastewater, industrial effluents, and hydrochloric acid derived from coal combustion^7–11^. Increasing chloride concentrations in lakes can change the acid neutralizing capacity of the water (pH), increase the transport and bioavailability of heavy metals^12^, increase lake stratification^13^, and alter ecological communities^4,14,15^, thereby degrading water quality and habitat.

Many long-term studies from around the world have identified anthropogenic-induced salinization of lakes, including many large freshwater bodies such as the Laurentian Great Lakes^7,16^, Lake Champlain^17^, Lake George^18^, Lake Constance^19^, and the deep subalpine lakes in Northern Italy^20^. To quantify the prevalence and drivers of long-term salinization in lakes worldwide, we collected chloride data from government organizations, universities, and lake associations; however, datasets covering at least ten years were only available in Europe and North America. The database presented here encompasses long-term chloride data from 529 lakes in Europe and North America, a geodatabase of lake polygon shapefiles, as well as a variety of lake characteristics, land cover metrics, and climate statistics. This database can be used to answer many questions about the salinization of freshwater lakes at different scales, and can be applied to address the impacts of salinization on the ecology of freshwater lakes^21^. Our hope is that other researchers will add additional lakes to expand the geographical coverage of our database so that the effects of lake salinization can be more easily addressed at a global scale.

## Methods

**Figure 1:**
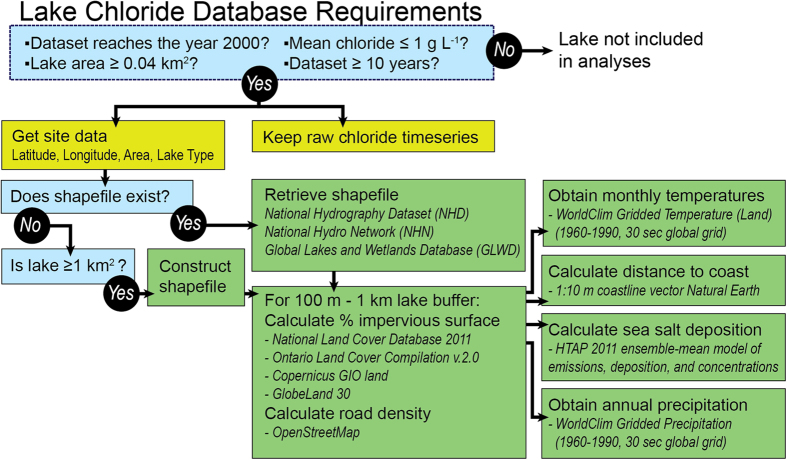
**Workflow for all datasets included in the global lake chloride database.**

The organizational workflow and data requirements for inclusion of lakes in the global lake chloride database are depicted in Fig. 1. All analyses were performed in the R statistical programming language^22^, using a suite of packages to access data and perform statistical analyses.

### Acquisition

Three primary approaches were used to collect lake chloride concentration data. Data were obtained from 1) online repositories containing datasets from multiple lakes, 2) online repositories containing data from single lakes, or 3) individual researchers, including members of the Global Lake Ecological Observatory Network (GLEON), and other organizations that responded to data requests. This range of approaches accounted for the multiple ways data were stored and shared, and as such, maximized the number of lakes included in our database.

For inclusion in this global lake chloride database, a site had to meet set criteria (Fig. 1).

1. The lake must have a surface area of at least 0.04 km^2^ (4 ha). This was the original size cutoff instituted by the United States Environmental Protection Agency (EPA) in their 2007 National Lakes Assessment.
2. The long-term mean chloride concentration must be less than 1 g l^−1^. This removes brackish and saline lakes from the database. Saline lakes are often defined as lakes with total dissolved solids>3 g l^−1^ (ref. 2).
3. The dataset must span at least ten years, and contain at least five data points. These criteria ensure both a robust measure of chloride concentrations and the ability to detect a long-term trend.
4. The dataset must include one chloride record after 2000. This criterion was established to ensure comparability in respect to time among site records.

**Figure 2:**
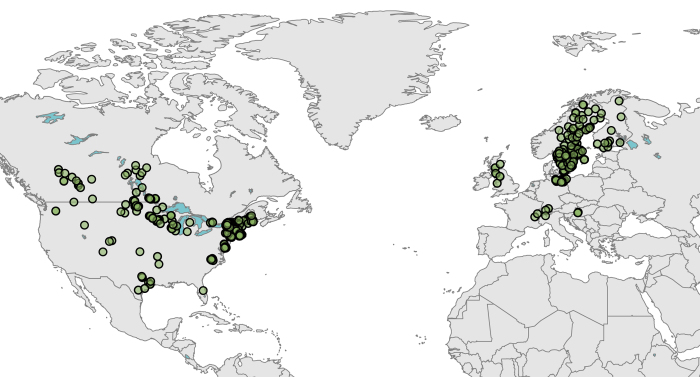
Map of North America and Europe showing the locations of lakes included in the global lake chloride database.

Once the data were collected, they were processed using an *a priori* metadata protocol. Each lake was assigned a project-specific identification number, and was paired with the associated data source name, source contact/acquisition information, and public accessibility designation (publicly available/private). We were unable to find any sites that met these criteria in South America, Africa, Asia, or Oceania. Many sites in the aforementioned regions had lake chloride measurements, but no records that spanned at least ten years, and therefore were not included in this database. In total, we collected 529 long-term datasets from ten countries (Fig. 2).

#### United States (*n*=315)

The majority of lakes included in this database are located in the United States (US). Data were obtained from the sources described below.

- The Water Quality Portal (WQP, http://www.waterqualitydata.us/) is a database that amalgamates data from federal, state, tribal, and local sources in the United States. It includes data from the EPA Storage and Retrieval (STORET) data warehouse and the United States Geological Survey (USGS) National Water Information System (NWIS). We searched the WQP for the characteristic name ‘chloride’ in ‘lakes, reservoirs, and impoundments’, using the dataRetrieval package in R (v2.5.5 (ref. 23)). Data extracted from the WQP included 67 lakes in Minnesota monitored by the Minnesota Pollution Control Agency. Lakes located in the Minneapolis/St Paul region are some of the most urban lakes included in the database (as determined by road density and land cover).
- North Temperate Lakes Long Term Ecological Research Site (LTER) collects long-term chloride data for eleven lakes in Wisconsin^24^. Data for Lake Wingra, Lake Monona, and Lake Mendota were augmented with earlier data from the Wisconsin Department of Natural Resources. In some cases, only annual averages were available; these sampling dates were labeled as July-01 (Lake Mendota: 1957–1972, Lake Monona: 1940–1987).
- Hubbard Brook LTER (http://www.hubbardbrook.org/) hosts 43 years of chloride measurements from Mirror Lake, New Hampshire.
- The Lake Champlain Basin Program provides a publicly accessible database of chloride measurements from Lake Champlain (http://www.lcbp.org/water-environment/data-monitoring/lake-and-watershed-data/). We obtained data for the Burlington Bay sampling site, and combined these with earlier data from USGS site 04295000. Lake Champlain (744 km^2^) is the only US lake represented in the ten largest lakes in our database.
- The EPA manages two long-term monitoring projects, specifically focused on the effects of atmospheric deposition into surface water bodies. These programs are the Temporally Integrated Monitoring of Ecosystems and Long Term Monitoring Project. The lakes included in these programs tend to be located in remote environments in New York, Vermont, Maine and New Hampshire.
- The New York City Department of Environmental Protection monitors chloride concentrations in all of its water supply reservoirs. Data were contributed from 10 reservoirs north of New York City. The location of sampling stations changed in 2006/07 in Boyd’s Corners, Cross River, Croton Falls, and Middle Branch Reservoirs.
- The University of Rhode Island Watershed Watch Program provided data for 59 lakes in Rhode Island (http://web.uri.edu/watershedwatch/uri-watershed-watch-monitoring-data/). Rhode Island lakes comprise some of the most urban lakes in the database.

#### Canada (*n*=37)

Alberta Environment and Sustainable Resource Development provided chloride data for eleven lakes in Alberta. The Water Quality Management Section of Manitoba Conservation and Water Stewardship provided chloride data for 16 lakes in Manitoba. Manitoba lakes represent four of the top ten largest lakes in the database, including Lake Winnipeg, the largest lake at 24,514 km^2^. Kawartha Conservation provided datasets exceeding 40 years for Balsam Lake, Cameron Lake, and Sturgeon Lake in Eastern Ontario. The International Institute for Sustainable Development provided data for four small lakes in the IISD Experimental Lakes Area (ELA) in northern Ontario. The Ontario Ministry of the Environment and Climate Change provided 15 years of data from Lake Simcoe. Of the ten largest lakes in the database, Lake Simcoe (722 km^2^) has the highest road density surrounding its shoreline. In 2008, the Ontario government introduced the Lake Simcoe Protection Act as a strategy to improve water quality.

#### Sweden (*n*=102)

A publicly available database of water chemistry was available through the Swedish Department of Water and Environment (http://info1.ma.slu.se/db.html). We included long-term monitoring data from a series of lakes that were designated as trend stations, which resulted in a collection of 101 lakes geographically distributed throughout the country. We also included data from data from Vänern, the largest lake in Sweden. Swedish coordinates were presented in the Swedish grid RT90 (ESPG:2400) at 10-m resolution, and were converted to WGS84.

#### Germany, France, and Switzerland (*n*=32)

Chloride data for Lake Constance were obtained from the Institut für Seenforschung in Langenargen. Data for Lake Zurich were provided by the City of Zurich Water Supply (WVZ) and the Amt für Abfall, Wasser, Energie und Luft (AWEL) of the Canton of Zurich, Switzerland. Chloride data for 28 lakes in Mecklenburg-Western Pomerania, Northern Germany were compiled for a review of German sulfate trends. All authorities and persons who supplied data are mentioned in the acknowledgements of Kleeberg^25^.

Data from Lake Geneva and Lake Bourget were accessed through the Alpine Lakes Observatory (SOERE OLA—Observation and Experimentation System for long-term Environmental Research). SOERE OLA is member of AnaEE-FRANCE and sponsored by AllEnvi, the National Research Alliance for the Environment. Physical, chemistry, and biodiversity data can be download using the information system developed by Eco-Informatique ORE team of the French National Institute for Agronomical Research (INRA, https://si-ola.inra.fr). INRA collects data from Lake Bourget with its partner CISALB, the intercommunal association of Lake Bourget, and Lake Geneva (also known as Lac Léman) with its partner CIPEL, the International Commission for the Protection of Leman.

#### Finland (*n*=11)

Water resource and environmental data for Finnish lakes are publicly available through the Finnish Environment Institute (SYKE) (http://www.syke.fi/en-US/Open_information). Chloride data for lakes included in our analysis were compiled by Lauri Arvola, at the University of Helsinki, through the online SYKE portal. Lakes Saimaa, Päijänne, and Inari are three of the largest lakes in the database; all lakes are over 1,000 km^2^.

#### United Kingdom (*n*=8)

The United Kingdom Upland Waters Monitoring Network (UK UWMN) is a consortium led by University College London (UCL), the NERC Centre for Ecology and Hydrology (CEH), Marine Scotland and Queen Mary College (QMUL). Originally funded by UK Government Department for Environment, Food and Rural Affairs, the network is currently supported by the partner organizations and a range of regional governments, agencies, and research institutes. The UK UWMN provided chloride data for eight UK lakes.

#### Hungary (*n*=3)

Water quality data are publicly available through Middle-Transdanubian and West-Transdanubian Water Directorates. We obtained long-term data from yearly reports of water quality of the Kis-Balaton Reservoir System and of Lake Balaton. Chloride concentrations were determined by argentometric methods according to Hungarian ISO Standards.

#### Italy (*n*=1)

The Institute of Ecosystem Study of the National Research Council (CNR ISE) in Verbania, Italy, provided chloride data for Lake Maggiore^20^. These data are collected in the framework of the long-term research program on Lake Maggiore funded by the International Commission for the Protection of Waters between Italy and Switzerland (CIPAIS). Lake Maggiore is the westernmost of the deep subalpine lakes in Northern Italy (LTER Europe site LTER_EU_IT_008). The lake area is 212.5 km^2^ and its maximum depth is 370 m.

### Chloride data

Each lake entry contains, at minimum, the sample date and the chloride concentration, converted to standardized units (mg l^−1^). Sampling depth was included for most sites. In some datasets, decadal gaps between sampling dates existed because early data points were sparse. Because trend analyses can be overly sensitive to outlying data points, we added additional quality control criteria; if a decadal gap was present in a dataset, at least five sampling points had to exist prior to the gap; otherwise, earlier data were not included. Additionally, the tsoutliers package in R (v0.6 (ref. 26)) was used to find and remove additive outlier data points, which although rare, skewed the linear and additive models’ fit to the data. The approach uses a t-statistic to test the significance of outliers at each time step and highlights those above a critical value. In our analyses, we conservatively chose a critical value of 20. In total, the data from 483 lakes were unchanged, and 64 lakes had 1–9 data points removed. The number of outliers removed from the original dataset is included in the metadata.

### Lake data

Each lake was identified by its latitude and longitude (in decimal degrees) and given a unique ID number. The country, or the province/state for North American lakes, was determined from the geographic coordinates. In the United States, the county was added to the file name, as there are often lakes with duplicate names within a state. Lake areas were obtained from a variety of published data sources. In rare cases, where lake area was not available, lake area was calculated from custom shapefiles in ArcGIS (see section on shapefiles). Lakes were classified as natural lakes or reservoirs. This distinction was made from visual inspection of satellite images for a dam, or from published government reports or articles that identified the waterbody as a reservoir. 407 of the 529 lakes had maximum lake depth data, in meters. The distance of the lake to the nearest coast (km) was calculated using the coordinates of the lake and a 1:10 m coastline vector shapefile available from Natural Earth (http://www.naturalearthdata.com/downloads/10m-physical-vectors/10m-coastline/), using the equal-area mollweide projection. The coastline vector was edited to exclude the St Lawrence Seaway.

### Climate data

To ensure homogeneity across regions, all climate data were obtained from gridded global datasets.

#### Temperature and precipitation

Monthly mean temperatures and annual precipitation totals were obtained using the WorldClim dataset, which contains high resolution global interpolated climate data from 1960–1990 (ref. 27). We used the highest resolution 30 s data, which is equivalent to 0.86 km^2^ at the equator. Lakes in the interior of North America, including those in North Dakota, Alberta, and Colorado receive less than 500 mm of precipitation a year. In contrast, some lakes in Vermont and the UK average greater than 1,300 mm a year.

#### Sea salt deposition

A global data set of wet and dry sea salt (NaCl) deposition was obtained from the World Data Centre for Precipitation Chemistry^28^ (http://www.wdcpc.org/assessment). We used the north grid of the HTAP 2001 ensemble-mean model results for emissions, deposition, and concentration, which has a spatial resolution of 1.0° latitude x 1.0° longitude^28^. Sea salt deposition is given as the combined total wet and dry deposition (kg ha^−1^ yr^−1^). Overall deposition was lower over North American lakes (mean=13 kg ha^−1^ yr^−1^), than European lakes (mean=38 kg ha^−1^ year^−1^). Deposition was highest in the United Kingdom, where lakes receive an average of 98 kg ha^−1^ of sea salt deposition each year.

### Shapefiles

Shapefiles that geographically delimited the perimeter of the lakes were required to quantify surrounding land use patterns. Shapefiles were acquired via five methods:

1. Lakes in the continental United States were included in the National Hydrography Dataset (NHD, *n*=336, http://nhd.usgs.gov/). The high resolution NHD data are available at 1:24,000-scale.
2. Shapefiles for lakes in Canada were downloaded from the 1:50,000 National Hydro Network (NHN).
3. Shapefiles for Swedish lakes were available through the Swedish Water Archives (*n*=102, http://www.smhi.se/klimatdata/hydrologi/sjoar-och-vattendrag/). We used surface water body layer version Vy_y_2012_2. Multiple shapefiles were merged to create a single shapefile for Vänern (pers. comm. B. Denfield).
4. Shapefiles for European lakes greater than 0.1 km^2^ were obtained from the Global Lakes and Wetlands Database (GLWD Level 1 and 2) (*n*=43, http://www.worldwildlife.org/pages/global-lakes-and-wetlands-database). Lakes are presented at a scale of 1:1,000,000.
5. Shapefiles for Lake Fenek (12.5 km^2^) and Lake Hidvegi (17.1 km^2^) in Hungary, and eleven other small European lakes in the UK, Germany, and Finland were not publicly available. These shapefiles were generated manually using ArcMap10.2.2.

The method by which shapefiles were acquired is included in the metadata as NHD, NHN, Sweden, GLWD, or Manual.

### Land cover

We constructed buffer zones surrounding each lake to approximate the anthropogenic influence of watershed and shoreline runoff of chloride into lakes. Buffer widths of 100, 200, 300, 400, 500, 1,000, and 1,500 m were chosen based on previous findings, such as those by Kelting *et al.*^29^, who found that >70% of total variation in chloride concentrations in Adirondack Park, NY could be explained by the road density in 320- to 1,280-m buffer zones. Regalado and Kelting^30^ used a 100-m buffer as an estimate of vehicular spray distance, and Read *et al.*^31^ used a 200-m buffer to understand local drivers on lake characteristics across the United States. We calculated both the percent impervious surface and total road density within buffer zones surrounding each lake as metrics for urban development. Buffer zones were constructed using the gBuffer function in the rgeos package in R (v0.3-19) (ref. 32) using local WGS84 Universal Transverse Mercator (UTM) zone projections.

### Impervious surface

Impervious surfaces are all impenetrable artificial surfaces, including roadways and parking lots (concrete and asphalt), and building roofs^33^. Since impervious surfaces prevent water infiltration into soils, increased runoff from these urban landscapes can threaten water resources^34^. Impervious surfaces have previously been linked to road salt application and the salinization of fresh waters^9,35,36^.

In land cover datasets, impervious surface is typically represented two ways: Method 1) a pixel is represented either as impervious or not-impervious (Boolean value), or Method 2) impervious surface is a continuous variable within each pixel from 0 to 100 percent^37^. The latter is a more robust quantification of impervious surface^37^.

#### United States

The 2011 United States National Land Cover Database (NLCD) is a national land cover product at a spatial resolution of 30 m (http://www.mrlc.gov/nlcd11_data.php). The NLCD presents the option of quantifying impervious surface via both methods from above. Method 1 is implemented via the NLCD 2011 Land Cover layer^38^. Impervious surface was defined as the total of all pixels classified as developed. Method 2 is implemented via the NLCD 2011 Percent Developed Impervious layer^39^.

#### Canada

A national land cover product was not available for Canada. Instead, we used two different products. For Ontario, we used the Ontario Land Cover Compilation v.2.0. (https://www.ontario.ca/data/land-cover-compilation). The OLCC has a 5-m pixel resolution. Impervious surfaces include settlements/infrastructure, transportation routes, and built-up areas. For Manitoba and Alberta, we used the LULC thematic raster data from GlobeLand 30 (http://www.globallandcover.com/GLC30Download/index.aspx) at a spatial resolution of 30 m. For both products, pixels are represented as a Boolean classification of impervious surface (Method 1).

#### Europe

For all European lakes, we used the European Space Agency’s GIO-land (Global monitoring for environment and security/Copernicus Initial Operations land) layers (http://www.eea.europa.eu/themes/landuse/gio-land). GIO-land includes a 20-m resolution layer of the percent impervious (1–100%), which enabled us to implement Method 2 for our calculation of impervious surface.

#### Road density

Road density has been used in previous studies as a proxy for urbanization and the application of road salt^29,40,41^. Road density was defined as the ratio of the length of the total road network in a given area (km) to the land area (km^2^). Worldwide road data were downloaded from OpenStreetMap via the osmar package in R (v1.1-7 (ref. 42)). In each buffer zone, we selected ‘ways’ tagged as highway, which included all primary, secondary, residential, and service roads. In our database, the highest road densities in a 500-m buffer were located around lakes in Minneapolis/St Paul, Minnesota and were up to 27 km km^−2^. Road density and percent impervious surface in buffer zones surrounding a lake were positively correlated overall (r^2^=0.69 in 500-m buffer, r^2^=0.79 in 1,500-m buffer); however, many lakes with low road density (0–5 km km^−2^) had 0% impervious surface.

## Data Records

The final database includes three files (Data Citation 1).

1. All descriptive lake data are formatted as a horizontal data table and is provided in chloride_concentrations.csv (Table 1).
2. Chloride time series data are formatted as a long data table and is provided in lake_characteristics.csv (Table 2).
3. Shapefiles for 529 lakes are provided as shapefiles in a zip file.

The database is designed so additional lake data can be easily added as they become available. All climate and land cover metrics were derived using open source datasets, and processed using open source tools. As new long-term datasets become available, or current datasets are expanded, the database can be updated with minimal time investment.

## Technical Validation

**Figure 3:**
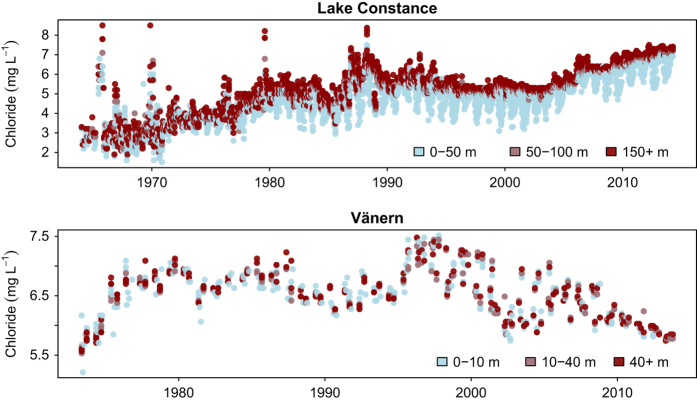
Time series of chloride concentrations in Lake Constance and Vänern. Data points are colored by lake depth.

For sites where maximum lake depth was not available, we have inferred that samples were collected at the surface, although this cannot be verified. Where samples were collected at multiple depths, we visually examined a selection of these lakes and found that chloride trends were consistent throughout the water column. For example, in Lake Constance and Vänern, both large freshwater lakes, chloride concentrations in the hypolimnion (bottom waters) and the epilimnion (surface waters) show similar trends through time (Fig. 3).

**Figure 4:**
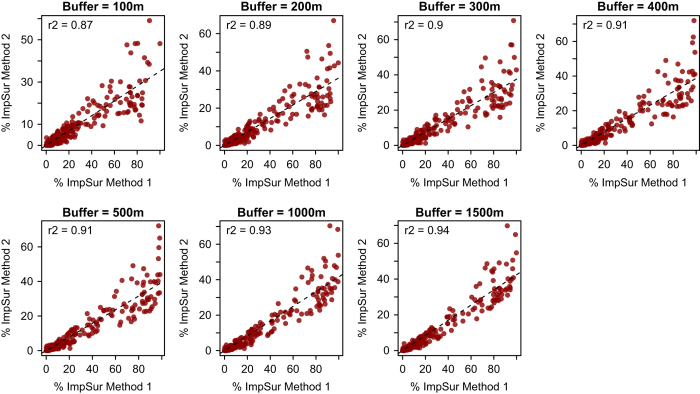
Comparison of the two methods of quantifying impervious surface in 100–1,500-m buffer zones around all US lakes in the database. In Method 1, each pixel is a Boolean value (TRUE, impervious; FALSE, not impervious). In Method 2, each pixel is quantified as percent impervious surface from 0–100%.

**Figure 5:**
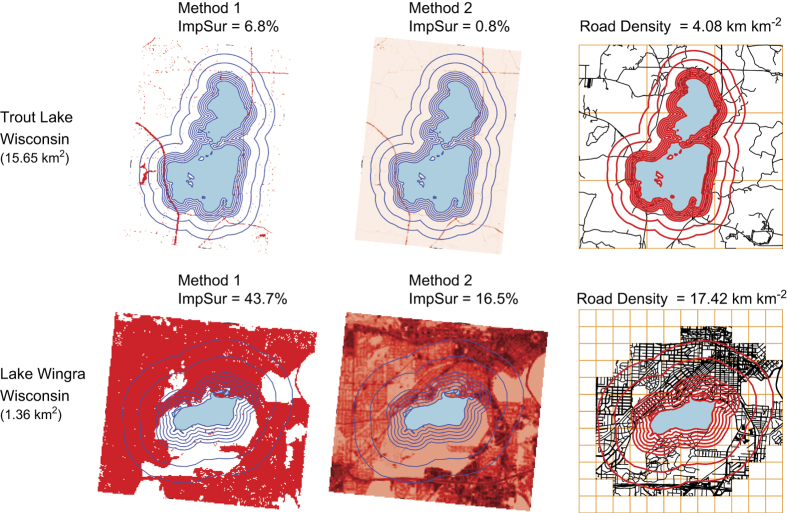
Comparison of impervious surface and road density calculations for Trout Lake and Lake Wingra, Wisconsin, USA. Values are given for a 500-m buffer. Impervious surface calculations via Method 1 have higher absolute values than Method 2, but relative differences are similar (see Fig. 4).

A comparison of the two methods used for calculating impervious surface reveal the measure of impervious surface surrounding a lake using Method 1 typically returns higher estimates, these are highly correlated to values calculated using Method 2, and become more tightly correlated as buffer width increases (Figs 4 and 5).

## Additional Information

**How to cite this article**: Dugan, H. A. *et al.* Long-term chloride concentrations in North American and European freshwater lakes. *Sci. Data* 4:170101 doi: 10.1038/sdata.2017.101 (2017).

**Publisher**’**s note**: Springer Nature remains neutral with regard to jurisdictional claims in published maps and institutional affiliations.

## Supplementary Material



## Figures and Tables

**Table 1:** Column names, column description, and percentage of sites with data for each feature for lake file.

**Column name**	**Description**	**Coverage**
SALT_ID	Internal lake ID number	100%
FileName	File name	100%
Common.Name	Name of lake or reservoir that is commonly used.	100%
Latitude (decimal degrees)	Latitudinal coordinate of lake	100%
Longitude (decimal degrees)	Longitudinal coordinate of lake	100%
Continent	Geographic location of lake	100%
Country	Geographic location of lake	100%
State	Abbreviation of state or province (only for North American lakes)	69.1%
Area.km2	Surface area of lake	100%
Depth.m	Maximum depth of lake	66.4%
LakeType	Type of lake: natural or reservoir	100%
YearMin	Start year of dataset	100%
YearMax	End year of dataset	100%
YearsTotal	Total years of dataset coverage	100%
MeanCl.mgL	Mean chloride concentration	100%
OutliersRemoved	Number of outliers removed from original data	100%
ShapefileMethod	Method used to obtain shapefile. Either: circle, GLWD, manual, NHD, or NHN.	100%
CoastDist.km	Distance to the nearest coastline	100%
WetDryDep	Sea salt deposition in kg ha^−1^ yr^−1^ (sum of wet and dry deposition)	100%
Precip.mm	Mean annual total precipitation from 1960 to 1990	100%
TempMMM	Long-term mean monthly temperatures from 1960 to 1990. MMM represents the month of the year.	100%
RoadDensityXXX	Road density, where XXX represents 100, 200, 300, 400, 500, 1,000, and 1,500-m buffer zones surrounding the lake. Measured in km km^−2^.	100%
LandImperviousBinXXX	Impervious surface, where XXX represents 100, 200, 300, 400, 500, 1,000, and 1,500-m buffer zones surrounding the lake. Calculated from datasets with binary pixels via Method 1 (see above). Measured in percent.	69.2%
LandImperviousPerXXX	Impervious surface, where XXX represents 100, 200, 300, 400, 500, 1,000, and 1,500-m buffer zones surrounding the lake. Calculated from datasets with percentage pixels via Method 2 (see above). Measured in percent.	92.9%

**Table 2:** Column name and description for lake chloride data file.

**Column Name**	**Description**
SALT_ID	Internal lake ID number
FileName	File name
Common.Name	Name of lake or reservoir that is commonly used
Station	Sampling station ID. The ID is program specific and is included to cross-reference sites with original sources. For some lakes, data are present from multiple stations.
Sample.Date	Date of sample collection. All data were collected on discrete days, with the exception of early data from Lake Mendota and Monona (see Methods).
Sample.Depth	Depth of sample collection
Chloride	Chloride concentration in mg l^−1^
Decimal.Date	Sample date provided as decimal value
Std.Chloride	Chloride concentration provided as a standardized z-score value, which was calculated for each lake
Integrated.Depth	Is TRUE if sample was integrated over a portion of the water column. Is FALSE otherwise. If a sample depth is given, this was the starting depth of sampling.
